# Retinal vascular occlusion and SARS-CoV-2 vaccination

**DOI:** 10.1007/s00417-022-05707-5

**Published:** 2022-05-25

**Authors:** Stela Vujosevic, Celeste Limoli, Simona Romano, Lucia Vitale, Edoardo Villani, Paolo Nucci

**Affiliations:** 1 Department of Biomedical, Surgical and Dental Sciences, University of Milan, Milan, Italy; 2 Eye Clinic, IRCCS MultiMedica, Milan, Italy; 3 University of Milan, Milan, Italy; 4 Department of Clinical Sciences and Community Health, University of Milan, Milan, Italy

**Keywords:** Retinal vascular occlusion, Retinal imaging, COVID-19, COVID-19 vaccination

## Abstract

**Purpose:**

To assess the clinical and retinal imaging features of patients in whom retinal vascular occlusion (VO) had developed in temporal associations with COVID-19 vaccination.

**Methods:**

In this retrospective case series, all consecutive adult patients with new onset VO within 6 weeks of vaccination against COVID-19 were included in the study between May 1 and October 31, 2021. All patients had a systemic medical health assessment, full ophthalmic evaluation, and complete fundus imaging.

**Results:**

Fifteen eyes of VO (14 patients) after COVID-19 vaccinations were identified. The median time between vaccination and symptoms onset was 14 days (range 7–42 days). The mean best-corrected visual acuity (BCVA) was 20/55 with a range of 20/20 to 20/200. Eleven of 15 eyes (73.3%) had visual acuity improvement after intravitreal treatment at 60–90 days (range, 45–105 days) from the presentation. Four of 5 cases without systemic risk factors for VO had a mean BCVA > 20/32 at presentation and > 20/25 at the latest evaluation.

Between May 1 and October 31, 2021, a temporal association was found between the 15 reported cases and COVID-19 vaccination out of a total of 29 VO (*p* = 0.05). The incidence of VO was higher in the considered period compared to the equivalent 6-month period in 2019 (1.17% vs 0.52%, respectively; *p* = 0.0134).

**Conclusions:**

Retinal vascular occlusion with different grades of severity are reported in temporal association with COVID-19 vaccination. The exact pathogenic mechanism needs to be further studied. No certain causal relationship can be established from this case series.

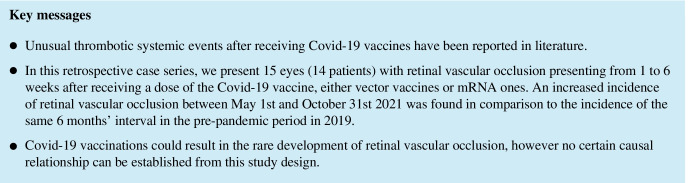



## Introduction

The development of vaccines against severe acute respiratory syndrome coronavirus 2 (SARS-CoV-2) infection has been shown to be the most important countermeasure to curb COVID-19 (coronavirus disease) pandemic. Vaccines have been reported to protect against severe illness from SARS-CoV-2 infection [1–5]. Thus far, four types of COVID-19 vaccines have been approved by the European Medicines Agency: mRNA vaccines, including BNT162b2, Pfizer-BioNTech, and mRNA-1273, Moderna; vector vaccines, including ChAdOx1 nCoV-19/AZD1222, Vaxzevria (ex-Oxford-AstraZeneca) [1–5] and Ad26.COV2, Janssen Johnson & Johnson.

The mass vaccination campaign against SARS-CoV-2 started in Italy on the 27th of December 2020. As of October 2021, the Italian Ministry of Health and Prevention announced that over 45 million people (83.5% of the population) had completed the recommended scheme and were fully vaccinated [6]. The mRNA vaccination is administered in 2-dose series separated by 3 weeks (21 days), the ChAdOx1 in 2-dose series separated by 8–12 weeks, whereas Ad26.COV2 has a single-dose regimen [6].

Given the scale of the current vaccination program, several rare ocular adverse events related to vaccines have been reported and their potential manifestations constitute a safety concern [7].

In this single-center case series, we hereby define the longitudinal characteristics of patients with retinal vascular occlusion (VO) in the temporal context of SARS-CoV-2 vaccinations, focusing on the time lapse between vaccination and disease onset, clinical and imaging features, and short-term outcomes.

A report of such ocular adverse events is timely and would be beneficial to design and implement protocols for close monitoring of patients that may be at higher risk.

## Methods

All consecutive adult patients with new onset retinal VO within 6 weeks of vaccination against COVID-19 who presented to the Medical Retina & Imaging Service at the University Eye Clinic San Giuseppe Hospital, Milan, Italy, between May 1, 2021, and October 31, 2021, were included in this retrospective case series study.

The diagnosis of VO was based on a comprehensive ophthalmic examination, best-corrected visual acuity determination (BCVA); slit lamp evaluation, intraocular pressure (IOP), dilated funduscopy, optical coherence tomography (OCT) and OCT angiography (OCT-A) (Heidelberg Spectralis HRA-OCT; Heidelberg Engineering, Heidelberg, Germany), ultra-wide field fundus color photo, and fluorescein angiography (FFA) (Optos California, Optos PLC, Dunfermline, UK).

Different types of VO had been diagnosed, including central retinal artery occlusion (CRAO), branch retinal artery occlusion (BRAO), and retinal vein occlusion (RVO), classified as central retinal vein occlusion (CRVO), hemiretinal vein occlusion (HRVO), and branch retinal vein occlusion (BRVO) [8]. Blood test including basic metabolic panel, complete blood count, coagulation tests like activated partial thromboplastin time (aPTT), thrombin time (TT) and prothrombin time (PT), C-reactive protein (CRP), erythrocyte sedimentation rate (ESR), fibrinogen, and homocysteine excluded thrombotic risk or underlying hematologic disease with possible influence on the onset of RVO.

ECG and carotid ultrasound were also performed to uncover any possible additional risk factors. In the younger patient, also factor V Leiden gene mutation, factor VIII activity, protein S activity, and antithrombin III (AT III), antinuclear antibodies (ANA), lupus anticoagulant, and anticardiolipin antibodies were tested.

No other concurrent ocular conditions were present at the time of the diagnosis. Patients provided proof of a negative COVID-19 test that met performance standards, either as an antigen or nucleic acid amplification tests [9].

Vaccination data by November 2021 including the delivered number of vaccine doses, types, and presence of ocular adverse event post COVID-19 vaccination in Italy are provided in Table 1 [7, 10].

**Table 1:** Vaccination data by November 2021 in Italy and presence of ocular adverse event post COVID-19 vaccination

Type of vaccine	Number of delivered doses	Ocular adverse event (total of reported adverse event) (%)
Total	99,797,303	1716 (114,279) (1.5%)
BNT162b2 (Pfizer)	71,173,035	2 (163) (1.2%)
mRNA-1273 (Moderna)	15,233,690	443 (31,272) (1.4%)
Ad26.COV2 (Janssen)	1,846,027	89 (4135) (2.2%)
ChAdOx1 (Vaxzevria)	11,544,551	1182 (78,709) (1.5%)

Data (through September 26, 2021) summarized from: Agenzia Italiana del Farmaco. Available at: https://bi.aifa.gov.it/SASVisualAnalyticsViewer/VisualAnalyticsViewer_guest.jsp?reportName=FVG_Intro0_report&reportPath=/Shared+Data/BI+FARMACOVIGILANZA/Public/Report/&appSwitcherDisabled=true [7]

Data on delivered vaccine doses were retrieved from https://www.governo.it/it/cscovid19/report-vaccini/ [10]

To investigate the possible temporal association between COVID-19 vaccines and VO, we counted and confronted the new cases of VO presenting to our Medical Retina & Imaging Service occurred within 6 weeks of vaccination and the cases occurred beyond 6 weeks in the inclusion period (May 1 to October 31, 2021). In addition, we compared the incidence of VO of this period to the equivalent period prior to the onset of COVID-19 pandemic (May 1 to October 31, 2019).

The study was approved by the Ethics Committee of IRCCS MultiMedica and adhered to the Tenets of the Declaration of Helsinki. Each patient gave informed consent for use of data.

### Statistical analysis

Categorical variables were reported with number and percentage, and continuous variables were summarized with mean and range. Univariate comparison between groups of VO patients was performed using the Fisher’s exact test. A log-linear analysis was applied to compare the proportions of the different categorical variables among the three groups, one-way analysis of variance (ANOVA) was applied to compare the means of numerical variables (age and BCVA) followed by Bonferroni post hoc analysis.

A *p* value $\le$ 0.05 was considered significant. The data analysis was performed using SPSS Statistics version 28 (IBM).

## Results

Fifteen cases of VO (14 patients) in temporal association with SARS-CoV-2 vaccinations (BNT162b2 *n* = 8, ChAdOx1 *n* = 6, Ad26.COV2 *n* = 1) were identified.

Six cases (40%) occurred after the first ChAdOx1 dose (Vaxzevria), 8 cases (53.3%) were noted following the second BNT162b2 dose (Pfizer), and 1 case (6.6%) after Ad26.COV2 (Janssen). No cases were reported after mRNA-1273 (Moderna). The median time between vaccination and symptoms onset was 14 days (range 7–42 days).

Characteristics of all 14 patients are presented in Table 2.

**Table 2:** Details on patients’ demographics, clinical features, treatment**,** and outcome

Patient No	Age (years)	Sex	Medical history	Vaccine type	Dose given	Time from vaccine to symptoms	Visual acuity at diagnosis of vasculits	Vascular occlusion features	Laterality	IOI	Edema	Hemorrhages	Ischemia	Neovascularization	Vitreous	Treatment	Visual acuity at latest evaluation
1	69	F	DVT	ChAdOx1	I	1 week	20/32	Infero-temporal BRVO	RE	-	-	+	Quadrant	No	No	Laser photocoagulation	20/20
2	82	F	None	BNT162b2	II	2 weeks	20/63	Infero-temporal BRVO	RE	-	+ + +	+	Quadrant	No	No	Steroid treatment	20/40
3	96	F	HTN T2DM	BNT162b2	II	1 week	20/200	CRVO	RE	-	+ + + , SND	+ +	No	No	No	Steroid treatment	20/200
4	91	F	None	BNT162b2	II	1.5 weeks	CF	CRVO	LE	+	+ + +	+ + +	No	No	No	Patient refused	CF
5	78	F	None	BNT162b2	II	1 week	20/25	Supero-temporal BRVO	RE	-	+	+	No	No	No	Anti-VEGF agents	20/20
5	78	F	None	BNT162b2	II	1 week	20/20	Supero-temporal BRVO	LE	-	-	+	No	No	No	None	20/20
6	70	M	None	ChAdOx1	I	1 week	20/20	CRVO	RE	-	-	+ + +	No	No	No	None	20/20
7	40	M	Hyperhomocysteinemia	ChAdOx1	I	2 weeks	20/20	BRVO	RE	-	-	+	No	No	No	None	20/20
8	76	M	HTN	ChAdOx1	I	6 weeks	CF	CRAO	RE	-	-	-	No	No	No	None	CF
9	91	M	T2DM	BNT162b2	II	4 weeks	20/32	BRVO	RE	-	+ + + , SND	-	No	No	No	Steroid treatment	20/32
10	72	F	HTN HL	BNT162b2	II	3 weeks	20/25	Supero-temporal BRVO	RE	-	+	+	Quadrant	No	No	Steroid treatment	20/20
11	88	M	HTN HL CVD Alzheimer K Prostate	BNT162b2	II	2 weeks	20/125	HRVO	RE	-	+ +	+	No	No	No	Steroid treatment	20/125
12	73	F	HTN HL CVD NET	ChAdOx1	II	4 weeks	CF	CRVO	RE	-	+	+ + +	No	No	Yes	Steroid treatment	CF
13	65	F	HTN HL T2DM	Ad26.COV2	I	1 week	20/40	CRVO	RE	-	+ , small SND	+ + +	No	No	No	Steroid treatment	20/32
14	72	F	HTN CVD	ChAdOx1	I	2 weeks	20/50	HRVO	LE	-	-	+ + +	Superior quadrants	No	No	Laser photocoagulation	20/50

*RE* right eye, *LE* left eye, *IOI* intraocular inflammation, *F* female, *M* male, *DVT* deep vein thrombosis, *HTN* hypertension, *T2DM* type 2 diabetes mellitus, *HL* hyperlipidemia, *CVD* cardiovascular disease, *NET* neuroendocrine tumor, *CRVO* central retinal vein occlusion, *HRVO* hemiretinal vein occlusion, *BRVO* branch retinal vein occlusion, *CRAO* central retinal artery occlusion, *CF* counting fingers, *SND* subfoveal neuroretinal detachment

In the present case series, women were affected slightly more often than men (9 (64.2%) versus 5 (35.7%), respectively); the median age was 77 years (range, 40 to 96).

Nine patients (64.2%) met the criteria for classic risk factors for RVO. More specifically, 7 (50%) patients had systemic hypertension (HTN), 4 (28.5%) had hyperlipidemia (HL), 3 (21.4%) had diabetes mellitus type 2 (T2DM), where 6 (42.8%) patients had 2 of these risk factors and 1 (7.1%) had all 3.

One patient (7.1%) had a previous ocular history of BRVO in the fellow eye. Four patients (28.5%) had negative medical history.

Unilateral acute vision loss was the most common presenting symptom; 1 patient (7.1%) presented with bilateral vision loss. The mean BCVA was 20/55 with a range of 20/20 to 20/200. The most severely affected eyes (patients 3, 4, 8, 12) had severe visual acuity loss with BCVA of 20/200 at the time of diagnosis and showed limited improvement at latest follow-up (range, 45–105 days). Four of 5 cases with no associated systemic risk factors for VO had a mean BCVA > 20/32 at presentation and > 20/25 at the latest evaluation.

A spectrum of severity of clinical findings found at presentation is outlined in Table 2.

VO included CRVO in 5 (33.3%) eyes, HRVO in 2 (13.3%) eyes, BRVO in 7 (46.6%) eyes, and 1 (6.6%) eye with central retinal artery occlusion (CRAO).

Treatment included administration of intravitreal antivascular endothelial growth factor (VEGF) in 1 (6.6%) eye, the use of a sustained-release intravitreal dexamethasone implant in 7 (46.6%) eyes, whereas 2 (13.3%) eyes required retinal laser photocoagulation due to extensive ischemia, not in association with anti-VEGF injections. Overall, 11 eyes (73.3%) had visual acuity improvement at 2–3 months (range, 45–105 days) from the presentation; however, 4 (26.6%) eyes did not achieve any BCVA recovery from the presentation. Although the follow-up was relatively short, there has been no recurrence of RVO.

In addition to the 15 cases of VO identified in temporal association with COVID-19 vaccination (< 6 weeks), other 14 cases of VO not related to vaccination (> 6 weeks) occurred, for a total of 29 cases of VO presented at our Medical Retina Service from May 1 to October 31, 2021. We found a statistically significant temporal association of the 15 reported cases with COVID-19 vaccination (*p* = 0.05), as they occurred within 6 weeks of vaccination and the remaining 14 cases occurred beyond that period (out of a total of 26 weeks).

Finally, between May 1 and October 31, 2021, there were 2470 cases visited at the Medical Retina Service, with an estimated incidence of VO of 1.17% cases. In the same temporal interval in 2019, there were 2671 cases of which 14 cases with VO (0.52%) visited at the same Medical Retina Service. The incidence of newly diagnosed VO was significantly higher in 2021 vs those in 2019, before the COVID-19 pandemic, *p* = 0.0134.

Tables 3 and 4 describe demographic data, clinical features, treatments, and outcomes of patients with VO in pre-pandemic period and those with VO occurred beyond 6 weeks from vaccination during the COVID-19 pandemic, respectively. No statistically significant differences were found in either studied clinical variables among the three different groups.

**Table 3:** Details on demographics, clinical features, treatment, and outcome of patients with VO in pre-pandemic period (2019)

Patient No	Age (years)	Sex	Medical history	Visual acuity at diagnosis of vasculits	Vascular occlusion features	Laterality	IOI	Edema	Hemorrhages	Ischemia	Neovascularization	Vitreous	Treatment	Visual acuity at latest evaluation
1	81	M	CVD HTN	20/200	CRVO	LE	-	+	+	+	No	No	Steroid treatment	20/50
2	81	F	None	20/40	Supero-temporal BRVO	RE	-	+	-	-	No	No	Anti-VEGF agents	20/63
3	73	M	HTN, HL	20/32	BRVO	LE	-	-	+	+	No	No	Laser photocoagulation	20/25
4	55	F	HTN	20/25	HRVO	LE	-	-	+ +	-	No	No	None	20/20
5	69	M	HTN, HL	CF	CRVO	RE	-	-	+	+	No	No	Laser photocoagulation	CF
6	72	M	Ulcerative colitis, antiaggregant therapy	20/40	Supero-temporal BRVO	RE	-	+ +	+	±	No	No	Anti-VEGF agents	20/25
7	62	F	HTN, antiaggregant therapy	20/50	HRVO	LE	-	+	+	+	No	No	None	Missing data
8	70	F	HTN	CF	CRVO	LE	-	+ +	+	±	No	No	Steroid treatment	CF
9	85	F	HTN, antiaggregant therapy	CF	CRVO	RE	-	+ +	+ + + +	+ + +	No	No	Steroid treatment	20/100
10	72	F	Antiaggregant therapy	20/25	BRAO	LE	-	-	-	+ +	No	No	None	20/20
11	83	F	CVD	CF	Supero-temporal BRVO	LE	-	-	+	+ -	No	No	Missing data	Missing data
12	79	M	HTN, T2DM	20/100	CRVO	RE		+ +	+	-	No	No	Anti-VEGF agents	20/100
13	81	M	HTN	Missing data	CRVO	RE	-	-	+	-	No	No	None	Missing data
14	72	F	Oral anticoagulant therapy	CF	CRVO	RE	+	-	+	-	No	±	None	20/100

*RE* right eye, *LE* left eye, *IOI* intraocular inflammation, *F* female, *M* male, *HTN* hypertension, *T2DM* type 2 diabetes mellitus, *HL* hyperlipidemia, *CVD* cardiovascular disease, *CRVO* central retinal vein occlusion, *HRVO* hemiretinal vein occlusion, *BRVO* branch retinal vein occlusion, *CRAO* central retinal artery occlusion, *CF* counting fingers

**Table 4:** Details on demographics, clinical features, treatment and outcome of patients with VO occurred beyond 6 weeks from vaccination during the COVID-19 pandemic

Patient No	Age (years)	Sex	Medical history	Visual acuity at diagnosis of vasculits	Vascular occlusion features	Laterality	IOI	Edema	Hemorrhages	Ischemia	Neovascularization	Vitreous	Treatment	Visual acuity at latest evaluation
1	75	F	HTN, HL, antiaggregant therapy	20/160	CRVO	RE	-	+ + +	+	+ +	No	No	Anti-VEGF agents, laser photocoagulation	20/160
2	89	F	HTN, oral anticoagulant therapy	20/32	Combined superotemporal BRAO + BRVO	RE	-	-	+	+	No	No	None	20/20
3	63	M	None	20/160	CRVO	LE	-	+	+	+ +	No	No	Anti-VEGF agents, laser photocoagulation	CF
4	61	M	None	20/20	BRVO	LE	-	-	-/ +	-	No	No	None	20/20
5	50	F	None	20/100	BRVO	LE	-	+	+ +	±	No	No	Steroid treatment	20/32
6	85	F	HTN, hypotiroidism	20/63	BRVO	LE	-	+ +	+	-	No	No	Anti-VEGF agents	20/63
7	80	F	None	20/32	Inferotemporale BRVO	RE	-	+ +	+	-	No	No	Steroid treatment	20/63
8	70	F	2TDM	20/16	BRVO	RE	-	+ +	+	-	No	No	Anti-VEGF agents	20/25
9	86	F	HTN, CVD	20/100	Superotemporal BRVO	LE	-	-	+	+ +	No	No	Laser	CF
10	58	F	HTN	20/25	Inferior BRVO	RE	-	Small SND	+	+	No	No	None	20/20
11	70	F	2TDM	20/160	CRVO	RE	-	+ + + , SND	+	-	No	No	Steroid treatment	20/160
12	86	F	HTN, breast cancer	20/32	HRVO	LE	-	-	+	-	No	No	None	20/32
13	49	F	HTN	20/25	CRVO	RE	-	-	+		No	No	None	20/20
14	75	M	2TDM	20/40	Supero-temporal BRVO	LE	-	-	+	+	No	No	Laser photocoagulation	20/25

*RE* right eye, *LE* left eye, *IOI* intraocular inflammation, *F* female, *M* male, *HTN* hypertension, *T2DM* type 2 diabetes mellitus, *HL* hyperlipidemia, *SND* subfoveal neuroretinal detachment, *CVD* cardiovascular disease, *CRVO* central retinal vein occlusion, *HRVO* hemiretinal vein occlusion, *BRVO* branch retinal vein occlusion, *CRAO* central retinal artery occlusion, *CF* counting fingers

## Conclusion

In this case series, we present 15 eyes (14 patients) with RVO and CRAO presenting from 1 to 6 weeks after receiving a dose of the COVID-19 vaccine, either mRNA vaccines or vector ones.

In the 6-month inclusion period (May 1, 2021–October 31, 2021), we found a temporal association between the 15 reported cases and COVID-19 vaccination out of a total of 29 VO. In addition, an increased incidence of VO between May 1 and October 31, 2021, was found in comparison to the incidence of the same 6-month interval in 2019. Therefore, these findings could foster the temporal association between COVID-19 vaccination and VO to explain the increase in the number of patients presenting with VO in the short period of time noted above.

The occurrence of ocular adverse events after SARS-CoV-2 immunization is rare, as reported on a monthly basis by AIFA in Italy [10], and is presented in Table 1. Retinal vascular adverse events after COVID-19 vaccinations appear to be rare [11]. However, there is now a growing literature reporting single case reports with RVO after both mRNA vaccines including mRNA-1273 (Moderna) [12] and BNT162b2 (Pfizer) [13–16], and vector-based ChAdOx1 (Vaxzevria) [17–19]. Other single case reports have documented combined CRAO-CRVO shortly after mRNA-1273 (Moderna) [20], combined CRAO-CRVO with ischemic optic neuropathy after BNT162b2 (Pfizer) [21], and CRAO after ChAdOx1 (Vaxzevria) [22].

Besides the reported ophthalmic cases, unusual thrombotic systemic events after COVID-19 vaccines, including ChAdOx1 nCoV-19 (Vaxzevria), Ad26.COV2.S (Janssen) [23–31], have increasingly been reported in literature. The condition is described as COVID-19 vaccine-related thrombosis and thrombocytopenia, namely thrombosis with thrombocytopenia syndrome (TTS).

Three separate case series have described patients who developed TTS after ChAdOx1 vaccination (Vaxzevria)[23–25]. TTS, which clinically simulate heparin-induced thrombocytopenia, is mediated by platelet-activating antibodies against platelet factor 4 (PF4) [26] and, interestingly, is more frequently observed following the first dose of ChAdOx1 (Vaxzevria) [23–25]. Furthermore, it has been noted that TTS manifest at 1- to 4-week period post-vaccination, which corresponds to the time for mounting a secondary antibody response [26, 27]. Similar hematological findings have been reported after Ad26.COV2 vaccination (Janssen) [28], which is an adenoviral-based vaccine like the ChAdOx1 (Vaxzevria).

To investigate the association between COVID-19 vaccines and hematological and vascular adverse events, a Scottish national population-based prospective cohort study has been conducted [29]. Positive associations were seen between the first-dose of ChAdOx1 (Vaxzevria) and idiopathic thrombocytopenic purpura as well as venous, arterial thromboembolic, and hemorrhagic events [29]. BNT162b2 (Pfizer), by contrast, did not show a statistically significant association with the aforementioned adverse events [29].

Interestingly, our findings of RVO occurrence after the first dose of ChAdOx1 are consistent with the results of these studies.

Therefore, it may be tempting to suggest a common pathogenic pathway of these thromboembolic events, linking the interaction of adenoviral-based vaccine vector versus SARS-CoV-2 with PF4 and other specific host proteins and the contribution to rare adverse events like RVO.

With regard to mRNA-based vaccines, it remains still unknown the pathogenic pathway underlying the observed vascular adverse events. Further studies are needed to investigate it and if there is any common pathogenic mechanism already described for ChAdOx1.

To the best of our knowledge, this is the first case series to report the temporal association between COVID-19 vaccination and VO. Our analysis showed that more cases were reported after mRNA-based vaccine Pfizer, followed by adenovirus vector-based DNA vaccines, Vaxzevria and Janssen. The lack of cases after mRNA-based vaccine Moderna could be deemed due to the smaller number of given doses in Italy at the time of the analysis [7]. However, similar levels of effectiveness and safety profile of both the mRNA-based vaccines were found in real-world use in Italy [10].

The clinical course of the VO had a range in the severity of findings; most patients responded well to therapy or recovered spontaneously, and visual acuity was preserved. Of note, 4 of 5 cases without risk factors for RVO had good final visual outcome.

VO presumably related to SARS-CoV-2 vaccination is an entity that apparently shares several features with the typical VO. These ocular findings seem to overlap with ophthalmic manifestations induced by COVID-19 itself, implying a common pathogenetic pathway between SARS-CoV-2 virus and vaccine-mediated immune response [30]. The pathogenesis of abnormalities in the retina subsequent to COVID-19 vaccinations could be explained by immunologic response elicited by components of the vaccine (spike antigen, other viral epitopes, human, or chimpanzee adenoviral components), and by molecular mimicry, where the vaccine components share structural similarities with self-antigens leading to an immunological self-tolerance break and an autoimmune response [31].

In this context, the pathogenic mechanism of VO after COVID-19 vaccination has not been elucidated and it is important to emphasize that no certain causality can be established from this case-series.

However, we need to acknowledge that the study was single-center, the design retrospective, and these results might also be attributed to other neglected variables, including the closure of private eye clinics diverting cases to our tertiary referral center during the pandemic and the likely increase of cardiovascular related events as an indirect effect of COVID-19 pandemic restrictions.

Finally, one important issue concerns the presumable time period of cause-effect relationship between vaccination and RVO. In this case series, this interval was set to 6 weeks, in reasonable accordance with available data and established knowledge linking vaccines with vascular or autoimmune conditions [32].

Considering the massive rollout vaccination campaign and the well-established excellent safety profile related to vaccines, it is important to highlight the very low incidence of reported adverse events in the literature. Consequently, COVID-19 vaccination should be strongly encouraged, having shown to be one of the most effective means to reduce the risk of getting and spreading the virus.

In conclusion, retinal vascular occlusion can be considered a rare manifestation of the spectrum of ophthalmic complications after COVID-19 vaccination. Patients with preexisting cardiovascular risk factors seem to be more likely to develop this complication, however further studies with more data are warranted to draw final conclusions about eventual association between VO and COVID-19 vaccinations.

Therefore, physicians should consider VO if patients present with vision loss within 6 weeks from COVID-19 vaccination.

Incidence of ocular adverse events after vaccination appears to be rare but should prompt a thorough ophthalmic evaluation.
